# Epithelioma of the Larynx

**Published:** 1907-06-29

**Authors:** 


### Points in Surgery.

### EPITHELIOMA OF THE LARYNX.

Without entering into any minute account of epithelioma of the larynx, there are one or two points in connection with it which seem worthy of discussion. The earliest symptom is, as a rule, some huskiness of the voice, usually without much pain, and the chief lesions from which it has to be diagnosed are:—(1) Paralysis of one vocal cord, such as might be caused by a thoracic aneurysm; (2) chronic catarrhal laryngitis; (3) tuberculous laryngitis; (4) syphilitic laryngitis.

The first step in the diagnosis is to make a laryngoscopic examination; vocal cord paralysis will at once be excluded, because in this affection the cords will be seen to be themselves clearly defined and healthy, except that one of them is either motionless on phonation, or at least moves less than does the other, whereas with epithelioma a structural alteration in the larynx will be present.

In the early stages it may be difficult to exclude catarrhal laryngitis, because when epithelioma is present there is nearly always some catarrhal trouble as well, and the epitheliomatous mass may at this time be comparatively small. The history may help one, however; the patient's occupation may necessitate excessive use of the voice; for example, he may be a clergyman, a business man who has to shout prices on 'change, a street hawker, or something of that kind; and the trouble with the voice may have been present for so long that malignant disease is very unlikely.

The two lesions which are most difficult of exclusion when epithelioma of the larynx is in question are tuberculous and syphilitic ulcerations of the larynx. There are, of course, many cases in which there is no difficulty at all in diagnosing tuberculosis laryngitis—the patient may have well marked physical signs of pulmonary phthisis to which the laryngeal trouble is obviously secondary. There are also many cases in which syphilis of the larynx is equally obvious. There remain, however, many others in which the diagnosis lies between the three things—tubercle, syphilis, and epithelioma—and it may be some time before the doctor can decide which of the three his patient is suffering from.

The appearances seen in the larynx at post-mortem examinations in these different affections are usually so characteristic—the multiple small ulcers with surrounding pallid œdema and absence of healing in tubercle—the big bilateral deep ulcers, with much destruction of the epiglottis and deformity by healing with scar-tissue in syphilis—the infiltrating, eroding, unilateral ulcer with fungating edges in epithelioma—that one might think that a laryngoscopic examination during life ought to settle the question at once. So it does in some cases; and if there is one point more than another upon which stress may be laid in distinguishing epithelioma from syphilis or tubercle in this way, it is that *epithelioma is invariably unilateral.* If one looks at the specimens preserved in the bottles on our hospital museum shelves, one sees case after case in which primary epithelioma of the larynx is sharply restricted to one side, the other vocal cord and its surroundings being perfectly healthy; whereas syphilis and tubercle, on the other hand, invariably affect both sides of the larynx at once. If one can see the parts so clearly with the laryngoscope that one can make out extensive ulceration on the one side and no ulceration at all upon the other, the condition is, with every probability, malignant. The difficulty is that there is often so much frothy mucous about and so much secondary œdema and even inflammation all round the growth that one is not always able to say that the trouble is unilateral.

The next step in the diagnosis would naturally be to make a very careful examination of the physical signs in the lungs, and also of the patient's sputum. The presence of abnormal physical signs at the apex of one or both lungs, suggestive of phthisis, would be presumptive evidence in favour of the laryngeal trouble being tuberculous. This would be further substantiated by finding tubercle bacilli in the sputum or in a swabbing from the larynx itself. On the other hand the absence of abnormal physical signs at the apex of one lung and the absence of tubercle bacilli from the sputum at one examination, do not exclude the possibility of tuberculosis of the larynx. It is quite true that tuberculous laryngitis as a primary affection is extremely rare; to all intents and purposes it is always secondary to tubercle in the lung; but it is an every-day experience to find no abnormal physical signs in the chest even when there is considerable phthisical mischief. The physical signs of pulmonary disease are only to be detected when the disease is at the surface of the lung; there may be quite big cavities at an apex and yet, if those are everywhere surrounded by healthy lung, they cannot be detected at all easily. In many cases it therefore seems as if there were primary tuberculous laryngitis, and later on the signs of tubercle develop in the chest; it is probable that in most of these instances there really was a primary focus in the lung, a secondary infection of the larynx from the sputum, and a tertiary re-infection of the lung from the larynx. Be this as it may, however, there is no doubt that the absence of abnormal lung signs does not greatly assist the diagnosis of epithelioma of the larynx, because even in the absence of signs of phthisis the laryngeal trouble may be tuberculous.

The modern physician would, no doubt, have the tuberculo-opsonic index of his patient's serum determined, with a view to seeing whether it indicated a liability to tuberculosis. If the index were normal (*i.e.*, 1) the evidence would not help him one way or the other; if, on the other hand, it were either low (*e.g.*, 0.5) or high (*e.g.*, 1.5), it would be an argument in favour of the presence of tubercle.

If, after the above examinations, the diagnosis cannot be decided, and syphilis appears to be a possibility either on account of the patient's history, or because of the appearance of the larynx, or because there are signs of syphilis in other parts of the body, a course of iodides and mercury should be tried; or else microscopical examination of a fragment of the laryngeal tissue should be undertaken at once. The effects of iodides and mercury in syphilis is to convert the granuloma material into fibrous tissue, with a resultant scar, so that it will entirely depend upon how far the trouble has already gone, how much relief these drugs will give in syphilitic cases. It is sometimes forgotten that iodides and mercury cannot replace a tissue that has been destroyed by syphilis. They will not bring back the epiglottis or the vocal cords if the syphilitic ulceration has destroyed them. All they will do is to cause the ulcers to heal, and convert the small round cells in the tissues round the ulcers into mature fibrous tissue; the scarring and deformity of the larynx will increase rather than diminish. Hence the results of giving these drugs often seem to be but slight, and their effects are neither so immediate nor so marked as in the case of lesions in the skin, which can be readily watched. Moreover, it is not uncommon for malignant ulceration to show temporary and misleading improvement for a short time after the administration of iodides is begun, so that it is often a month or more before this method of arriving at the diagnosis tells one way or the other. Malignant disease of the larynx is, curiously enough, a notable exception to the general rule in respect to the age of malignant cases. It has occurred in a boy of three years old; no less than 8 per cent. of all the cases are under 30 years of age; and quite a big proportion are under 40.

If there are secondary deposits anywhere they are most likely to be in the cervical glands; general dissemination of epithelioma from the larynx to the liver and other organs is quite rare.

There remains the question of microscopical examination of a fragment snipped off from the affected region. It is not altogether a simple thing to obtain this fragment, unless one is an adept in the use of intra-laryngeal instruments; but, having obtained it, it would be expected that the microscope must necessarily clear up the diagnosis at once. Frequently this is the case, no doubt, but there are a few words of warning necessary. If the microscopical section of the fragment removed from the larynx shows typical epithelioma the diagnosis is decided at once; but there are two sources of fallacy; in the first place, it is comparatively easy to mistake for epithelioma the thickened and proliferated layers of squamous epithelial cells which result from underlying chronic inflammation, particularly if the section obtainable is but a small one. In the second place, and, more important still, the tissues at the margins of an epithelioma become inflamed and may readily seem part of the growth itself; the fragment obtained by intralaryngeal removal may very easily be some of this inflamed and bulging non-malignant tissue, with the result that the pathologist may report that he finds no sign of cancerous trouble in the part submitted to him for examination. Barwell has recently recorded a case of this kind, in which a successful excision showed that the patient had an undoubted epithelioma of the larynx deeply infiltrating the thyroid cartilage, but previous to excision, when a very considerable portion of a mass which bulged into the larynx and caused dyspnœa had been snipped away to free the air-way, microscopical examination of the part thus removed showed only normal tissues without a trace of neoplasm.

It is very largely the difficulties of a diagnosis that add interest to its making, and it is the difficulties we have discussed which add interest to the diagnosis of epithelioma of the larynx. If we may pick out three points deserving particular attention they are:—

(1) That primary epithelioma of the larynx is almost invariably unilateral.

(2) That it occurs at a much earlier age than is the rule with carcinomata in general.

(3) That the evidence afforded by microscopical examination of a fragment obtained by intralaryngeal removal is sometimes not so final as might be supposed.

